# Circulating and tumor-infiltrating immune cell profiles in cervical cancer checkpoint blockade: multi-omics biomarkers of response and resistance

**DOI:** 10.3389/fimmu.2026.1925452

**Published:** 2026-09-08

**Authors:** Wangshu Li, Xiaohui Yu, Wenjuan Wei, Yan Wang, Sitong Zhang, Aziz ur Rehman Aziz, Daqing Wang, Chunfang Ha

**Affiliations:** 1 Key Laboratory for Early Diagnosis and Biotherapy of Malignant Tumors in Children and Women, Dalian Women and Children’s Medical Group, Dalian, China; 2 Department of Gynecology, Ningxia Medical University General Hospital, Yinchuan, China

**Keywords:** biomarkers, cervical cancer, circulating immune cells, immune checkpoint blockade, single-cell sequencing, spatial proteomics, spatial transcriptomics, tumor-infiltrating lymphocytes

## Abstract

Immune checkpoint blockade has changed the therapeutic landscape of cervical cancer, but clinical benefit remains heterogeneous and difficult to predict with single biomarkers. Cervical cancer is a particularly suitable setting for immune-cell profiling because HPV-driven antigenicity, chemoradiotherapy-induced tissue remodeling, anti-angiogenic combinations, and repeated exposure to PD-1/PD-L1 inhibitors all shape the blood-tumor immune axis. This Mini Review discusses how circulating immune cells and tumor-infiltrating immune cells can be jointly interrogated by multi-omics technologies to identify biomarkers of response and resistance. We focus on single-cell RNA sequencing, T-cell receptor/B-cell receptor (TCR/BCR) profiling, cellular indexing of transcriptomes and epitopes by sequencing (CITE-seq), flow cytometry, spatial transcriptomics, high-plex spatial proteomics, bulk transcriptomics, cytokine profiling, and circulating tumor DNA (ctDNA)-informed longitudinal designs. Recent cervical cancer atlases suggest that response is unlikely to be explained by total immune infiltration alone, but instead reflects the spatial organization and functional states of cytotoxic T cells, NK cells, antigen-presenting cells, tertiary lymphoid structure (TLS) programs, regulatory T cells, myeloid cells, and cancer-associated fibroblasts (CAFs). We propose a blood-to-tumor framework in which dynamic peripheral phenotypes may monitor systemic immune competence, while tissue multi-omics may identify local immune exclusion, terminal exhaustion, and myeloid suppression. Prospective paired sampling and interpretable, externally validated AI-assisted computational integration are needed before these biomarkers can guide treatment selection.

## Introduction

Cervical cancer remains a major global malignancy despite vaccination and screening, with the highest burden in settings where prevention and access to multimodality treatment remain uneven (1). For recurrent, persistent, metastatic, and high-risk locally advanced disease, immune checkpoint blockade has moved from late-line activity to randomized phase 3 evidence. Pembrolizumab combined with chemotherapy, with or without bevacizumab, improved outcomes in persistent, recurrent, or metastatic cervical cancer; cemiplimab improved survival after platinum-based chemotherapy; pembrolizumab added to chemoradiotherapy improved progression-free survival in newly diagnosed high-risk locally advanced disease; atezolizumab plus bevacizumab and chemotherapy improved outcomes in the first-line advanced setting; and durvalumab with chemoradiotherapy did not meet its primary endpoint in CALLA (2–8). Additional phase 2 studies of PD-1 blockade in anti-angiogenic and neoadjuvant combinations further emphasize that the immunotherapy context in cervical cancer is expanding beyond a single line of treatment (9–11).

However, the biomarker question remains unsettled. PD-L1 combined positive score, tumor mutational burden, and conventional immune infiltration measures are clinically useful but incomplete, partly because they compress a dynamic immune ecosystem into static and often single-compartment variables (12–14). Existing reviews generally survey broad cervical cancer immunotherapy biomarkers or the tumor immune landscape (15, 16). In contrast, this Mini Review specifically links longitudinal circulating immune-cell monitoring with single-cell and spatially resolved tumor immune architecture to examine response and resistance to immune checkpoint blockade.

Cervical cancer also differs from many other solid tumors because viral oncogenes can support immune recognition while chronic HPV infection, local mucosal tolerance, stromal remodeling, and previous pelvic radiotherapy can promote immune escape. The increasing use of immune checkpoint blockade with chemotherapy, bevacizumab, radiotherapy, and tyrosine-kinase inhibitors further complicates biomarker interpretation: a marker may reflect PD-1 reinvigoration, chemotherapy-induced antigen release, vascular normalization, radiation-related lymphocyte injury, or a mixture of these effects. For this reason, a Mini Review on cervical cancer immunotherapy as a whole would be too broad. The more actionable unit is a blood-to-tumor immune profile tied to immune checkpoint blockade and to the specific clinical problem of response versus resistance.

## Literature selection

For this narrative Review, relevant peer-reviewed studies were identified through targeted PubMed searches and reference-list screening through August 2026 using combinations of “cervical cancer,” “immune checkpoint blockade,” “peripheral blood,” “single-cell,” “spatial transcriptomics,” “TCR,” “multi-omics,” “spatial proteomics,” “CODEX,” “imaging mass cytometry,” “artificial intelligence,” “response,” and “resistance.” Preprints and unpublished observations were excluded. Representative evidence is summarized in **Table 1**.

**Table 1:** Representative evidence supporting candidate blood- and tissue-based biomarkers. ICB, immune checkpoint blockade.

Study	Sample/treatment context	Platform	Candidate immune feature	Interpretation/limitation	Evidence origin
Li et al. (17); Liu et al. (18); Cao et al. (19)	Cervical tumor tissue; atlas cohorts	Single-cell RNA sequencing	Immune and stromal heterogeneity	Generates biomarker hypotheses; no direct ICB-response validation	Cervical-specific; descriptive
Ou et al. (20); Fan et al. (21); related spatial studies (24–26)	Cervical tumor tissue	Single-nucleus, spatial, and multi-omics profiling	Spatial ecosystems and fibroblast-associated exclusion	Mechanistic relevance; limited treated samples	Cervical-specific; mainly descriptive
Gong et al. (30); Dofutsu et al. (31)	Peripheral blood during cervical cancer ICB	Longitudinal immunophenotyping/lymphocyte counts	Dynamic immune subsets and lymphocyte reserve	Primarily prognostic; observational or retrospective	Cervical-specific; ICB-treated
Bai et al. (32)	Advanced or recurrent cervical cancer	Blood indicators and T-cell subsets	On-treatment systemic immune status	Associated with efficacy; external validation required	Cervical-specific; treated
Lan et al. (38)	Advanced cervical cancer; immune rechallenge	Spatiotemporal immune profiling	Evolution of systemic and local immune states	Rechallenge-specific; prospective replication required	Cervical-specific; treated
Aung et al. (43)	Non-small cell lung cancer receiving immunotherapy	Spatial multi-omics	Spatial immune signatures	Outcome-associated; requires cervical cancer validation	Other cancer; extrapolated
Xu et al. (55)	Advanced cervical cancer; chemotherapy, anti-angiogenic therapy, and PD-1 blockade	Tissue multi-omics/single-nucleus profiling	T-cell activity and B-cell/TLS programs	Response correlates in a selected single-arm cohort	Cervical-specific; treated
Schurch et al. (56); Giesen et al. (57); Rivest et al. (58); Phillips et al. (59)	Tumor and immune tissues; technology demonstrations	CODEX/Phenocycler, imaging mass cytometry, and automated seqIF (COMET)	Protein-defined immune states and spatial cell neighborhoods	High-plex protein localization; cervical ICB validation remains limited	Cross-tumor/technology; extrapolated

## Tumor-infiltrating immune cell states resolved by single-cell spatial omics

Spatial transcriptomics maps gene-expression programs within intact tissue architecture, whereas spatial proteomics localizes and quantifies proteins and immune-cell phenotypes *in situ*. Together, these complementary approaches resolve the organization, functional states, and cellular interactions of tumor-infiltrating immune cells within the tumor microenvironment. Recent single-cell and spatial studies have substantially refined the cervical cancer tumor immune microenvironment. Single-cell transcriptomic atlases show that malignant epithelial states, immune subsets, stromal populations, and intercellular communication programs vary across patients and histologic contexts (17–19). Spatial transcriptomics and single-nucleus approaches add a key missing dimension by mapping where immune cells, cancer cells, and stromal barriers co-localize within tumor regions (20). Multi-omic analyses of cervical squamous cell carcinoma further indicate that epithelial cell states can be linked to distinct immune ecosystems, including immune-enriched programs, cytokeratin/stromal programs, and cancer-associated fibroblast interactions that may contribute to immune exclusion (21). Studies comparing HPV-related histologic contexts and disease stages also support the view that immune architecture is not uniform across cervical cancers (22, 23). More recent spatial datasets have highlighted differences between tumor leading-edge and core regions, metabolic niches, SPP1-positive macrophage programs, and fibroblast-associated immune remodeling (24–26). Spatial transcriptomics and spatial proteomics should be distinguished rather than grouped as a single “spatial” modality. Spatial transcriptomics maps RNA programs and inferred cell states, whereas high-plex spatial proteomics directly quantifies protein-defined phenotypes and cell-cell neighborhoods. Platforms such as CODEX/Phenocycler, imaging mass cytometry (IMC), and automated sequential immunofluorescence on the Lunaphore COMET platform can resolve TCF1/TCF7, TOX, CD39, checkpoint receptors, lineage markers, and suppressive myeloid-stromal niches *in situ* (56–58).

These studies support three practical messages for biomarker development. First, the number of tumor-infiltrating lymphocytes is less informative than their functional state. Cytotoxic CD8+ T cells may represent active antitumor immunity, transient reinvigoration, or terminal exhaustion depending on co-inhibitory receptor expression, proliferative state, TCR clonality, and spatial access to tumor cells. Second, myeloid and stromal compartments can dominate resistance even in tumors that are not immune-desert. SPP1-positive macrophages, antigen-presenting defects, fibroblast-derived TGF-beta programs, extracellular-matrix barriers, and suppressive metabolic niches can separate T cells from malignant cells. Third, spatial organization is itself a biomarker. A tumor with activated T cells trapped at the invasive margin may behave differently from a tumor with direct immune-tumor contact, intratumoral dendritic cells, B-cell aggregates, or tertiary lymphoid structures.

T-cell exhaustion deserves particular attention because PD-1 blockade does not rescue every PD-1-positive T cell. Exhaustion is a differentiation continuum rather than a binary state. Precursor-exhausted CD8+ T cells are typically TCF1/TCF7-positive, retain proliferative and self-renewing capacity, and can expand after PD-1 blockade. More terminally exhausted states show loss of TCF1/TCF7, increased TOX and CD39, broader inhibitory-receptor co-expression, and reduced proliferative and reinvigoration capacity (27, 28). These differentiated populations may nevertheless retain cytotoxic molecules and short-term effector function. At the same time, metabolic stress, hypoxia, nutrient competition, and chronic antigen exposure can constrain CD8+ T-cell persistence and effector function (29). A tissue biomarker panel for immune checkpoint blockade should therefore capture both T-cell competence and the suppressive context in which those T cells are embedded.

At the cell-state level, response-oriented tissue variables should include effector CD8+ and NK-cell cytotoxicity, antigen presentation, dendritic-cell maturation, CXCL9/CXCL10-mediated immune recruitment, B-cell/TLS programs, and TCF1/TCF7-positive precursor-exhausted CD8+ T cells rather than predominantly terminally exhausted states. Resistance-oriented tissue variables should include SPP1+ tumor-associated macrophages, monocyte-derived macrophages with angiogenic or matrix-remodeling programs, immunosuppressive regulatory T cells, dysfunctional dendritic cells, cancer-associated fibroblast (CAF)-derived TGF-beta signaling, hypoxia or lactate-rich metabolic niches, and combinations of inhibitory ligands. Single-cell data define these cell states, but spatial data determine whether the states are arranged in a way that permits tumor killing. In a small biopsy, the key question is not simply whether the tumor is inflamed; it is where the effector cells are located, which cells they contact, and what suppressive neighborhoods surround them.

## Circulating immune-cell profiles during immune checkpoint blockade

Peripheral blood is not a substitute for tissue, but it is the most practical compartment for longitudinal immune monitoring. In cervical cancer, dynamic peripheral immunophenotyping has been associated with prognosis during immune checkpoint blockade, and peripheral lymphocyte count has been explored as a marker of outcome in patients receiving immune checkpoint inhibitors (30, 31). A prospective 2026 study further supports the feasibility of using peripheral blood indicators and T-cell subsets to evaluate immunotherapy efficacy in advanced or recurrent cervical cancer (32). These studies are not yet sufficient to define a standard blood biomarker, but they show why serial blood sampling should be embedded into cervical cancer immunotherapy trials. Absolute lymphocyte count (ALC) and neutrophil-to-lymphocyte ratio (NLR) are inexpensive but highly nonspecific systemic measures; current cervical cancer evidence supports their use mainly as prognostic or treatment-context markers rather than ICB-specific predictive biomarkers.

Findings from broader immunotherapy cohorts provide a useful framework. Peripheral blood-based biomarkers can include absolute lymphocyte count, neutrophil-to-lymphocyte ratio, monocyte or myeloid-derived suppressor cell (MDSC) abundance, effector-memory CD8+ T cells, proliferating Ki-67+PD-1+ CD8+ T cells, activated NK cells, regulatory T cells, cytokine signatures, TCR repertoire changes, and immune-related toxicity-associated phenotypes (33–37). These measures should be distinguished conceptually: prognostic biomarkers associate with outcome irrespective of treatment, pharmacodynamic measures change after ICB and reflect treatment exposure, and predictive biomarkers identify differential benefit from ICB; evidence for the latter remains limited in cervical cancer.

Importantly, the strongest rationale for blood profiling is its complementarity with tissue. A patient may have an inflamed tumor biopsy but poor systemic lymphocyte reserve; another may have activated circulating CD8+ cells but stromal exclusion in tumor tissue. Emerging work on immune rechallenge in advanced cervical cancer illustrates the value of spatiotemporal immune assessment, because resistance and renewed sensitivity may depend on how systemic and local immune states evolve over time (38). Thus, circulating biomarkers should be interpreted as dynamic readouts of systemic immune readiness rather than stand-alone surrogates for the tumor microenvironment.

Blood profiling also has important limitations. Peripheral inflammation can reflect infection, anemia, corticosteroid exposure, nutritional status, tumor burden, or radiation field rather than tumor-specific immunity. In cervical cancer, pelvic chemoradiotherapy can deplete lymphocytes, whereas chemotherapy, anti-angiogenic therapy, and tyrosine-kinase inhibitors can alter antigen release, vascular trafficking, and myeloid composition. Therefore, blood biomarkers should be modeled relative to treatment timing and baseline condition. A clinically tractable blood panel may include only a small set of robust variables: lymphocyte recovery, CD8+ T-cell activation or proliferation, regulatory T-cell frequency, suppressive myeloid abundance, NK-cell activation, selected inflammatory cytokines, and TCR dynamics. Chemotherapy can transiently reduce lymphocytes while altering antigen release and myeloid composition; pelvic chemoradiotherapy may cause deeper or prolonged lymphopenia, whereas anti-angiogenic therapy can modify immune trafficking. Longitudinal analyses should therefore align sampling with treatment cycles, quantify nadir and recovery, and avoid attributing treatment-induced lymphodepletion to ICB resistance without adjustment.

## Integrating blood and tissue multi-omics: from association to biomarker models

The central challenge is integration. Blood and tumor measurements answer different biological questions: blood reflects systemic immune reserve, inflammatory tone, clonal circulation, and treatment-induced perturbation, whereas tissue reflects antigen presentation, local suppression, spatial exclusion, and immune-tumor contact. Multi-omics approaches can link these dimensions, but they also introduce batch effects, sparse data, platform dependence, and overfitting risk (39–41). For a cervical cancer Mini Review context, the clinically relevant aim is not to use every technology for every patient, but to define which combinations can move a biomarker from discovery to trial validation.

**Figure 1:**
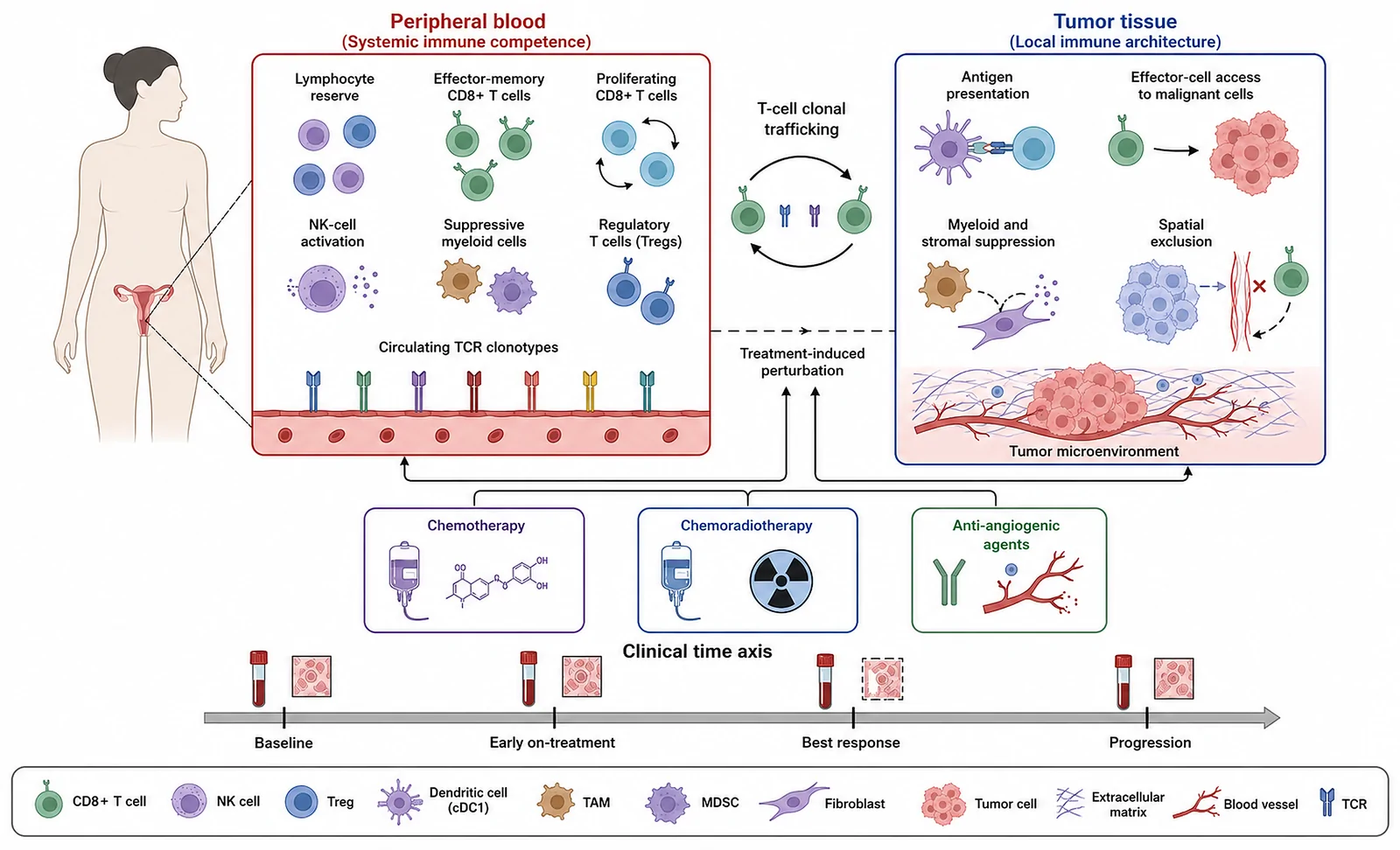
The blood-to-tumor immune axis in cervical cancer immune checkpoint blockade. Peripheral blood reflects systemic immune competence, whereas tumor tissue represents local immune architecture. These compartments are connected by T-cell clonal trafficking and are dynamically influenced by chemotherapy, chemoradiotherapy, and anti-angiogenic agents. The clinical time axis indicates blood and tissue sampling at baseline, early on-treatment, best response, and progression, with dashed outlines denoting optional biopsy. TCR, T-cell receptor; Treg, regulatory T cell; cDC1, conventional type 1 dendritic cell; TAM, tumor-associated macrophage; MDSC, myeloid-derived suppressor cell.

A practical discovery design would pair pretreatment tumor biopsy and blood, early on-treatment blood, optional on-treatment biopsy, and progression biopsy. Tissue assays can include single-cell or single-nucleus RNA sequencing, TCR/BCR sequencing, CITE-seq, multiplex immunofluorescence, spatial transcriptomics, spatial proteomics, bulk RNA sequencing, and targeted proteomics. Blood assays can include high-dimensional flow cytometry or mass cytometry by time of flight (CyTOF), PBMC single-cell profiling, plasma cytokines, circulating proteins, TCR clonotype tracking, and ctDNA dynamics. Analytical integration should preserve the clinical time axis: baseline, early treatment, best response, and resistance (**Figure 1**).

Computational methods can help convert high-dimensional data into smaller biomarker candidates. Spatial multi-omics in other tumor types has identified immune-cell neighborhoods, tumor-stromal proximity, and immune-exclusion patterns associated with immunotherapy outcomes; these concepts require cervical cancer-specific validation (42, 43). Machine learning can integrate images, transcriptomes, and clinical variables, but models must be interpretable, pre-specified, and externally validated before they can guide treatment (44, 45). Public resources and pan-cancer TME frameworks, including single-cell databases and microenvironment subtype models, can support hypothesis generation, but cervical cancer-specific validation remains essential because HPV biology, radiotherapy exposure, and anti-angiogenic combinations create disease-specific immune pressures (46–48). AI can integrate spatial transcriptomic and proteomic neighborhoods, digital pathology, longitudinal blood-cell and TCR trajectories, and treatment context to derive compact biomarker panels. A credible development pathway requires locked feature definitions, explicit handling of missing modalities, leakage control, calibration, interpretable outputs, and external validation against simpler clinical baselines before treatment selection is attempted.

Two levels of integration are especially relevant. The first is biological integration: linking a circulating clone or immune-cell state to a spatially mapped intratumoral counterpart. This can test whether an expanding peripheral TCR clonotype is entering tumor tissue, accumulating at the invasive margin, or merely reflecting bystander activation. The second is clinical integration: linking immune features to regimen, response assessment, progression pattern, and immune-related adverse events. A model that predicts radiographic response but also identifies early hyperinflammation or immune-related toxicity risk would be more useful than a tissue-only score. Multi-omics should reduce clinical uncertainty rather than simply multiply unvalidated features.

In addition, assay selection should be staged. In the discovery phase, broad single-cell and spatial platforms are valuable because they can reveal unexpected immune states and cell-cell interactions. In the validation phase, those discoveries should be compressed into reproducible measurements that can be applied to formalin-fixed tissue, routine blood samples, or archived trial specimens. In the clinical phase, the final assay should answer a practical question: whether to use immune checkpoint blockade, how to combine it, whether early progression reflects primary resistance, and which suppressive axis may be targetable at relapse.

## Response and resistance: candidate immune-cell profiles for clinical translation

A response-enriched profile in cervical cancer immune checkpoint blockade would likely combine systemic and local features rather than rely on one marker. Candidate blood features include preserved or recovering lymphocyte count, lower suppressive myeloid burden, expanding effector-memory or proliferating CD8+ T cells, activated NK cells, and TCR clonotypes shared between blood and tumor. Candidate tissue features include spatially accessible CD8+ T cells, antigen-presenting dendritic cells, IFN-gamma and chemokine programs, B-cell/TLS activity, and preserved antigen presentation. TLS biology is increasingly associated with immunotherapy responsiveness across cancers and deserves specific evaluation in cervical cancer tissues, especially in trials combining immune checkpoint blockade with chemotherapy, radiotherapy, or anti-angiogenic therapy (49, 50).

By contrast, a resistance-enriched profile is likely more heterogeneous. Tumors may resist immune checkpoint blockade through terminal T-cell exhaustion, loss of antigen presentation, stromal exclusion, suppressive myeloid cells, regulatory T cells, metabolic deprivation, hypoxia, or compensatory checkpoints. Contemporary reviews of the tumor microenvironment and myeloid-cell checkpoints emphasize that response to PD-1/PD-L1 blockade is shaped by macrophages, monocytes, neutrophils, dendritic cells, and stromal barriers, not only by T cells (51–53). In cervical cancer, chemoradiotherapy-induced remodeling can also create later resistance states; for example, ACKR2-positive tumor-cell programs have been linked to CD8+ T-cell senescence and recurrence after chemoradiotherapy (54), but they have not been shown to predict ICB response or resistance and require direct testing in ICB-treated cohorts. These findings support biomarker panels that explicitly measure suppressive myeloid/stromal programs and therapy-induced immune dysfunction.

**Figure 2:**
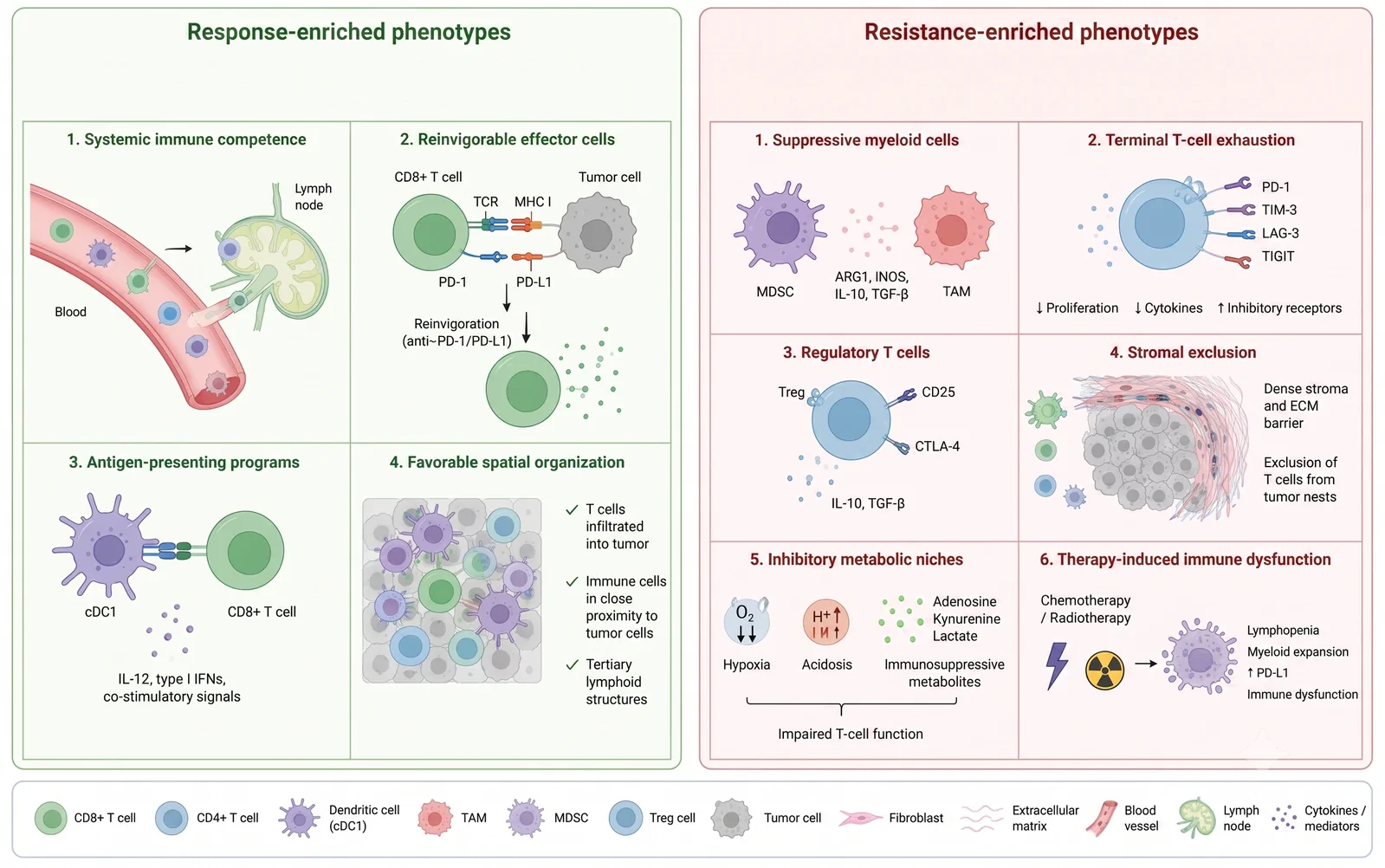
Candidate immune-cell profiles of response and resistance. Response-enriched phenotypes include systemic immune competence, TCF1/TCF7-positive precursor-exhausted CD8+ T cells with proliferative and reinvigoration capacity, antigen-presenting programs, and favorable spatial organization. Resistance-enriched phenotypes include suppressive myeloid cells, terminally exhausted T-cell states characterized by loss of TCF1/TCF7, increased TOX/CD39 and inhibitory-receptor co-expression, reduced proliferative capacity, regulatory T cells, stromal exclusion, inhibitory metabolic niches, and therapy-induced immune dysfunction.

Recent translational cervical cancer studies point toward the kind of integrated biomarker that may be needed. Molecular and immune correlates of response to de-escalated chemotherapy plus penpulimab, an anti-PD-1 antibody, and anlotinib, a multi-target anti-angiogenic tyrosine kinase inhibitor, identified tissue immune features, T-cell activity, B-cell/TLS programs, and multi-omic correlates that may explain unusually high response in a selected advanced-disease cohort (55). Such data do not establish a universal biomarker, but they illustrate a useful direction: response should be modeled as a coordinated immune state across blood, tumor, and treatment context. **Figure 2** summarizes candidate response- and resistance-enriched profiles.

These candidate profiles should not be interpreted as binary labels. A tumor can show both response and resistance features, for example high CD8+ infiltration together with SPP1+ macrophage niches, or TLS-like B-cell aggregates together with CAF-mediated exclusion. The translational task is to identify which axis is dominant and actionable. A T-cell-inflamed but myeloid-suppressed tumor may motivate evaluation of PD-1 blockade plus myeloid modulation; a stromal-excluded tumor may motivate evaluation of anti-angiogenic, TGF-beta, or matrix-remodeling strategies; and a lymphocyte-depleted patient may require careful sequencing around chemoradiotherapy. This argues for biomarker signatures that output a mechanism, not only a probability of response.

## Discussion and outlook

For clinical translation, the field should separate discovery-level multi-omics from deployment-level biomarker assays. Discovery studies can use single-cell, spatial, proteomic, and longitudinal blood profiling to identify immune states. Trial-validation studies should then compress these signatures into deployable assays, such as limited flow-cytometry panels, multiplex immunofluorescence, targeted RNA signatures, or combined blood-tissue scores. Clinical implementation will require pre-specified cutoffs, independent validation cohorts, reproducible computational pipelines, and regimen-specific interpretation. Spatial transcriptomics and spatial proteomics are complementary rather than interchangeable: the former supports discovery of RNA-defined programs, whereas the latter can translate selected protein states and neighborhoods into FFPE-compatible validation assays.

Several technical priorities are immediate. First, paired blood and tissue sampling should be built into cervical cancer immune checkpoint blockade trials whenever feasible. Second, analysis should account for timing, because chemotherapy, radiation, and anti-angiogenic therapy can remodel immune states before response is measurable radiographically. Third, spatial metrics should be reported in addition to cell abundance, including immune-tumor distance, lymphoid aggregate maturity, myeloid-stromal neighborhoods, and tumor-leading-edge features. Fourth, studies should report enough metadata to distinguish HPV status, histology, disease stage, previous radiotherapy, anti-angiogenic exposure, corticosteroid use, and antibiotic exposure when possible. Finally, models should be clinically interpretable. A complex multi-omics classifier has limited value if it cannot be reduced to a robust clinical decision rule.

Several gaps remain. Most cervical cancer single-cell and spatial atlases are cross-sectional, include limited numbers of treated samples, and often lack paired peripheral blood. Many trials also rely on archival tissue rather than fresh pretreatment biopsies, which can obscure the immune state present at treatment initiation. Published cohorts differ in histology, HPV type, PD-L1 scoring, previous radiotherapy, anti-angiogenic exposure, antibiotic exposure, and corticosteroid use. Without harmonized clinical metadata, multi-omics results may be biologically plausible but difficult to validate. Standardized sampling windows and common immune-cell annotation frameworks are therefore as important as new algorithms.

In conclusion, cervical cancer immune checkpoint blockade requires biomarkers that reflect both systemic immune competence and local tumor immune architecture. Circulating immune profiles offer feasible longitudinal monitoring, while tumor-infiltrating immune and stromal profiles explain the spatial and functional constraints that determine whether PD-1/PD-L1 blockade can restore antitumor immunity. The most promising path is a blood-to-tumor multi-omics framework that may distinguish response as coordinated immune activation and resistance as a measurable combination of immune exhaustion, myeloid suppression, and spatial exclusion.
